# In Silico Analysis of the Ga^3+^/Fe^3+^ Competition for Binding the Iron-Scavenging Siderophores of *P. aeruginosa*—Implementation of Three Gallium-Based Complexes in the “Trojan Horse” Antibacterial Strategy

**DOI:** 10.3390/biom14040487

**Published:** 2024-04-16

**Authors:** Nikoleta Kircheva, Stefan Dobrev, Vladislava Petkova, Lyubima Yocheva, Silvia Angelova, Todor Dudev

**Affiliations:** 1 Institute of Optical Materials and Technologies “Acad. J. Malinowski”, Bulgarian Academy of Sciences, 1113 Sofia, Bulgaria; nkircheva@iomt.bas.bg (N.K.); sdobrev@iomt.bas.bg (S.D.); vpetkova@iomt.bas.bg (V.P.); sea@iomt.bas.bg (S.A.); 2 Faculty of Chemistry and Pharmacy, Sofia University “St. Kliment Ohridski”, 1164 Sofia, Bulgaria; lyubima_d_dasheva@abv.bg; 3 University of Chemical Technology and Metallurgy, 1756 Sofia, Bulgaria

**Keywords:** Ga^3+^/Fe^3+^ competition, siderophore, pyochelin, pyoverdine, *Pseudomonas aeruginosa*, ESKAPE, maltolate, transferrin, DFT

## Abstract

The emergence of multidrug-resistant (MDR) microorganisms combined with the ever-draining antibiotic pipeline poses a disturbing and immensely growing public health challenge that requires a multidisciplinary approach and the application of novel therapies aimed at unconventional targets and/or applying innovative drug formulations. Hence, bacterial iron acquisition systems and bacterial Fe^2+/3+^-containing enzymes have been identified as a plausible target of great potential. The intriguing “Trojan horse” approach deprives microorganisms from the essential iron. Recently, gallium’s potential in medicine as an iron mimicry species has attracted vast attention. Different Ga^3+^ formulations exhibit diverse effects upon entering the cell and thus supposedly have multiple targets. The aim of the current study is to specifically distinguish characteristics of great significance in regard to the initial gallium-based complex, allowing the alien cation to effectively compete with the native ferric ion for binding the siderophores pyochelin and pyoverdine secreted by the bacterium *P. aeruginosa*. Therefore, three gallium-based formulations were taken into consideration: the first-generation gallium nitrate, Ga(NO_3_)_3_, metabolized to Ga^3+^-hydrated forms, the second-generation gallium maltolate (tris(3-hydroxy-2-methyl-4-pyronato)gallium), and the experimentally proven Ga carrier in the bloodstream—the protein transferrin. We employed a reliable in silico approach based on DFT computations in order to understand the underlying biochemical processes that govern the Ga^3+^/Fe^3+^ rivalry for binding the two bacterial siderophores.

## 1. Introduction

The emergence of multidrug-resistant (MDR) microorganisms combined with the ever-draining antibiotic pipeline poses a disturbing and immensely growing public health challenge [1,2,3], listed by the World Health Organization as one of the top ten threats, whose effective solution requires a multidisciplinary approach [4]. Notably, several bacteria, termed as ESKAPE species (*Enterococcus faecium*, *Staphylococcus aureus*, *Klebsiella pneumoniae*, *Acinetobacter baumannii*, *Pseudomonas aeruginosa*, and *Enterobacter* species) endanger humanity’s well-being to the greatest extent, most frequently causing nosocomial infections [5,6,7]. Their treatment is time-consuming, requires a combined scheme of two or more drugs, often exerts severe adverse effects, and hence the urgent necessity of novel therapies has been highly underscored in recent years [8,9]. Therefore, bacterial iron acquisition systems and bacterial Fe^2+/3+^-containing enzymes have been identified as a plausible target of great potential for the development of novel antimicrobial agents given the vital demand of the pathogen for iron for their fast proliferation, growth, and metabolism [10,11]. Its role resides in the interconversion between the ferrous (Fe^2+^) and ferric (Fe^3+^) oxidation states, which is accompanied by an electron transfer easily accomplished under physiological conditions. When evading the host, pathogens exert a high demand of iron, reaching up to 10^−6^ M in concentration [12]. As a means of withholding this essential component along with other metal cationic species of paramount significance, the host (higher) organisms have developed “nutritional immunity” [13], thus decreasing the extracellular iron concentration down to 10^−18^ M. In order to sequester iron effectively, pathogens have developed counter-mechanisms to those utilized by the recipient, the most interesting one being the excretion of small molecules denoted as siderophores (Greek for “iron” and “bearer”[14]), “pirating” iron from the surrounding media [15,16,17,18].

The novel and intriguing antibacterial approach aiming at depriving microorganisms from the essential iron, known as the “Trojan horse” strategy [10,19,20,21], combined with studies of “abiogenic”/“alien” metal ions (with no known function in the body but exerting a therapeutic effect under specific medical conditions) [22,23,24,25,26,27] has led to the recognition of gallium’s potential in medicine as an iron mimicry species. Nowadays, it is already known that it possesses antineoplastic, anti-inflammatory, immunomodulating, antihypercalcemic, and analgesic activities [28,29,30,31,32]. Its antibacterial properties, nonetheless, have attracted the greatest attention recently, especially since Ga-based compounds exert a well-pronounced antibacterial action against some ESKAPE microorganisms or *Mycobacterium* representatives [33,34,35,36,37,38,39]. Specific physicochemical characteristics, such as oxidation state (III), analogous coordination pattern (preferentially forming octahedral over tetrahedral complexes), and similar ionic radii in the corresponding structures [27,40], allow the “alien” cation to act as an iron mimetic species and iron-competitor for binding key proteins, mostly those related to the Fe^3+^ metabolism. 

**Figure 1:**
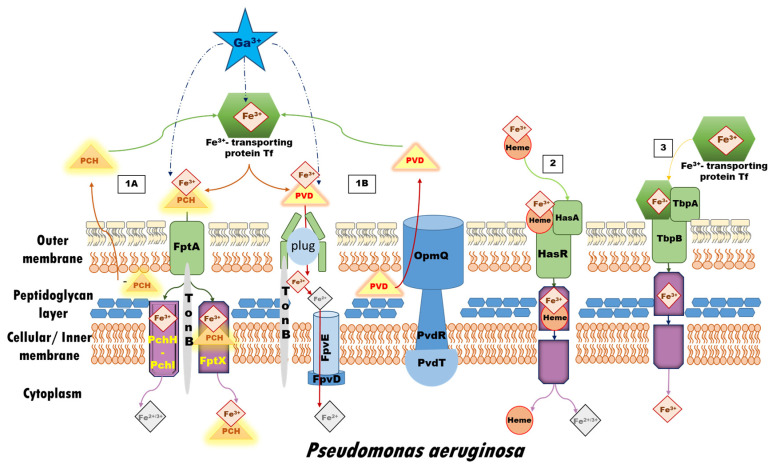
Iron up-take systems in *P. aeruginosa* in correspondence to the scope of the present research, and possible ways for gallium’s interference according to its initial form: Pathway 1 represents the iron-siderophore access: (1A) The Fe-PCH complex is recognized and further internalized into the cell by the FptA receptor, a specific TonB-dependent outer membrane transporter. A fraction of Fe-PCH dissociates in the periplasm through an unknown mechanism, and the free ferric/ferrous ions are further transferred into the bacterial cytoplasm across the inner membrane via the PchH-PchI pathway, while another fraction of the complex is carried by the FptX into the cytoplasm; (1B) The Fe-PVD complex is imported into the cell through FpvA, a specific TonB-dependent membrane protein transporter for iron-loaded pyoverdines. Following the conformational changes in the FpvA transporter, the ferric ion is released through a still unknown process but most probably involving reduction of the metal without degradation or chemical modification of PVD. The FpvCDEF ABC transporter is expected to participate in the dissociation of iron. Additionally, the PvdRT-OpmQ pump in *P. aeruginosa* effluxes back the ‘bare’ siderophore or other metal-PVD complexes into the intracellular space. Pathway 2 depicts the Heme-internalization system typical for Gram–negative bacteria, the HasA-type hemophores, capable of acquiring Heme from a broad range of host hemoproteins, including hemoglobin, hemopexin, and myoglobin. Although the step-by-step process is still under debate, it is widely accepted that HasA acquires Heme without direct interaction with the hemoproteins of the invaded host. Pathway 3 illustrates the Fur-regulated bipartite system consisting of an outer membrane receptor protein, TbpA, and a surface-associated lipoprotein, TbpB, both binding transferrin in its holo-form (metal-loaded), while TbpA is able to bind the apo-form as well. In contrast to Pathways 1 and 2, ferric ions must first be liberated from transferrin prior to uptake by TbpA. This figure is created in accordance with data provided in Ref. [61].

The current study focuses on the bacterium *P. aeruginosa***,** defined by the World Health Organization as a “Priority 1: Critical” pathogen in need of new approaches to treatment [4,41], since it is shown to be susceptible to gallium-based therapy [42]. It possesses the characteristics of a Gram-negative bacterium: a thin peptidoglycan layer, compensated by the presence of an additional outer membrane (differing from the inner one by its structure), unlike Gram-positive bacteria, which are distinguished by the thickness of their cell wall [43]. In correspondence to the scope of the present research, the main iron acquisition routes in *P. aeruginosa* and some pathways where gallium is hypothesized and/or accepted to be able to interfere in the iron(III) intra- and intercellular cycle (without claiming to be exhaustive) have been schematically depicted in the following Figure 1 [44]. Firstly, the iron-transport protein transferrin is revealed to be the main carrier of Ga^3+^ ions in the bloodstream, although it is unable to compete with the native one for binding in its active site, as gallium’s association constants at physiological pH come as the second after the ones for the ferric ion: log K_1_ = 19.75 and log K_2_ = 18.80 [45]. Note that other trivalent metal cations bind transferrin with much lower affinity, e.g., for the Cr^3+^ cation the corresponding log K_1/2_ values are 10.2 and 5.31 [46]. Furthermore, gallium’s ability to bind bacterial siderophores and enter bacterial cells through active transport in the form of Ga^3+^-siderophore complexes was studied by both experimental [47,48,49,50] and theoretical [51,52] approaches, thus providing evidence of gallium’s aptitude for implementation in the “Trojan horse” strategy. Once inside the cell, the alien ion binds some iron-requiring bacterial enzymes of great significance, such as ribonucleotide reductase [28,53,54], aconitase [38,39,55,56], catalase [36,57,58,59], and super-oxide dismutase [57,58,60]. 

**Figure 2:**
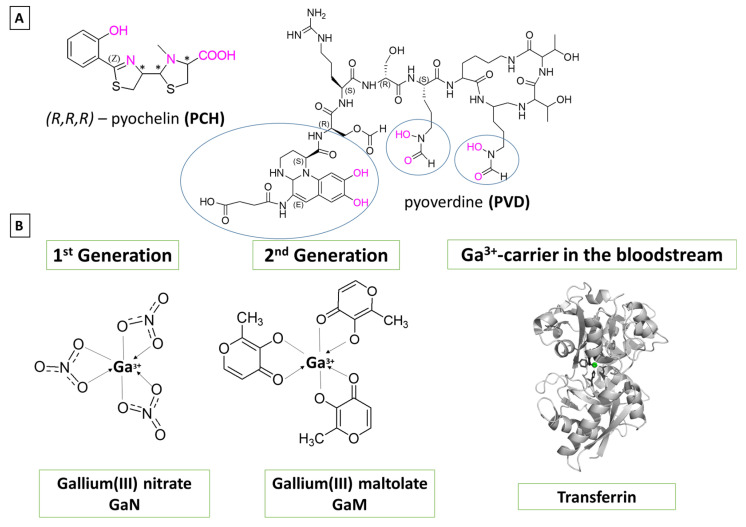
Chemical structures of (**A**) the two iron-scavenging *P. aeruginosa* siderophores pyochelin (denoted as PCH) and pyoverdine (denoted as PVD). The metal-ligating groups are colored in pink; (**B**) the two generations of gallium(III)-based potential drugs, gallium nitrate (GaN) and gallium maltolate (GaM), and the Ga^3+^-carrier in the bloodstream, transferrin, with its metal-binding groups.

The aim of the current study is to distinguish characteristics of immense significance in regard to the initial gallium-based complex allowing the alien cation to effectively compete with the native iron for binding the Fe^3+^-acquiring siderophores pyochelin and pyoverdine secreted by the bacterium *P. aeruginosa* [62]. Their chemical structures have been depicted in Figure 2A. The zinc-scavenging pseudopaline has not been considered here as it falls beyond the scope of the present research. Three gallium-based formulations were taken into consideration: the first-generation gallium nitrate, Ga(NO_3_)_3_, metabolized to Ga^3+^-hydrated forms depending on the pH of the surrounding media, the second-generation gallium maltolate (tris(3-hydroxy-2-methyl-4-pyronato)gallium), and the experimentally proven Ga carrier in the bloodstream—the protein transferrin modelled as the metal-binding amino-acid residues from its active site. Their structures are further depicted in Figure 2B. As it is of immense significance to understand the underlying biochemical processes that govern the Ga^3+^/Fe^3+^ rivalry for binding bacterial siderophores, we employed a reliable in silico approach proven effective when assessing the outcome in metal competition [63,64,65,66,67,68,69]. The differentiation in action between the three different formulations of the “therapeutic” abiogenic ion is accomplished by modeling all reactions of substitution with an attacking reagent (Figure 2B), being either one of the Ga^3+^-containing compounds. The obtained results draw a clear picture of gallium’s potential as an alternative antibacterial drug and aim to reveal novel aspects in its application in the “Trojan horse” strategy.

## 2. Materials and Methods

### 2.1. Reactions Modeled

The envisioned Ga^3+^-Fe^3+^ substitution reactions have been modeled as follows:

[Ga(OH)_4_(H_2_O)_2_]^−^ + [Fe_Sid]^n^ + H_2_O → [Ga_Sid]^n^ + [Fe(OH)_3_(H_2_O)_3_]^0^ + OH^−^ (R1);

[Ga(OH)_3_(H_2_O)_3_]^0^ + [Fe_Sid]^n^ + H_2_O → [Ga_Sid]^n^ + [Fe(OH)_2_(H_2_O)_4_]^+^ + OH^−^ (R2);

[GaM_3_]^0^ + [Fe_Sid]^n^ → [Ga_Sid]^n^ + [FeM_3_]^0^ (R3);

[Ga_Tf]^2−^ + [Fe_Sid]^n^ → [Ga_Sid]^n^ + [Fe_Tf]^2−^ (R4).

The first two reactions correspond to a probable attack from the metabolites of gallium(III) nitrate in two media with different pH values: at physiological pH of around 7 (R1), and at an acidic pH of about 5 (R2), where the hydrated metal complexes are consistent with the pKa values of Ga^3+^ (pKa_1_ = 3.09, pKa_2_ = 3.55, pKa_3_ = 4.4, and pKa_4_ = 6.05) and Fe^3+^ (pKa_1_ = 2.68, pKa_2_ = 3.73, pKa_3_ = 6.33, and pKa_4_ = 9.35) [70]. The lower pH corresponds to data provided in the literature suggesting that at sites of *P. aeruginosa* infection pH drops to acidic values, which is not only specific for this bacterium but also contributes significantly to its virulence [71]. The [Fe/Ga_Sid]^n^ structures present siderophore-bound metal cations in different ratios that have previously been reported in the literature (see below). Since pyochelin binds the metal cations in a tetradentate fashion, the preferable octahedral coordination of Fe^3+^/Ga^3+^ requires two more positions to be accommodated/fulfilled [72]. Therefore, two 1:1 metal:PCH complexes have been modeled with additional molecules from the solvent (expected to be water as the reactions take place in the intercellular space), but also in correspondence to the strong Lewis acidity of the metal cations (deprotonation of H_2_O leads to the formation of OH^−^ in the first coordination shell of the metal). The 1:1 metal:PCH complexes have hence been modeled as [Me_PCH_2W]^+^/[Me_PCH_2OH]^−^, where Me stands for either Fe^3+^ or Ga^3+^. The authors in Ref. [49] provide further strong evidence for the formation of a 1:2 metal:PCH complex, where one molecule pyochelin binds the metal in a tetradentate fashion, whereas the other one binds in a bidentate mode. These complexes have additionally been modeled as [Me_2PCH]^−^. Notably, our computations reproduce correctly bond length in these constructs with respect to the deposited metal-containing X-ray structures (see 2.3. Calibration and Validation of the applied DFT Procedure). Pyoverdine, on the other hand, provides six ligating groups, forming a stable metal:siderophore complex in a 1:1 ratio denoted as [Me_PVD]^−^. However, the implementation of DFT requires some restrictions in regard to the size of the system; hence, a simplified model for this siderophore reduced mostly to the metal-ligating groups has been applied (circled in the presented Figure 2A structure where the amino acid chain ends with a methyl group; see Supplementary Materials, Figure S1). A probable competition with the native ferric ions, where the second-generation gallium-based potential drug gallium maltolate [GaM_3_]^0^ is considered as an attacking reagent, has been taken into account in reaction (R3), leading to the formation of the corresponding Me^3+^-containing complexes. Reaction 4 (R4) depicts another possible pathway for a gallium attack, where the carrier of the abiogenic ion is the protein transferrin known to transport Ga^3+^ in the bloodstream, thus yielding iron-bound transferrin after the metal exchange. Its two identical metal-binding centers comprise a histidine, an aspartic acid and two tyrosines from the amino acid sequence of the protein, and an additional anion not part of the AA chain—most probably a carbonate (Figure 2B). The protein side chains have, therefore, been modeled as the neutral imidazole, an acetate anion, CH_3_COO^−^, and phenolate, C_6_H_5_O^−^, respectively (Figure 2C).

### 2.2. Computational Protocol

The applied in silico analysis is based on DFT calculations performed with the Gaussian 09 suite of programs [73]. The well-calibrated and validated (see 2.3. Calibration and Validation of the applied DFT Procedure) B3LYP/6-31+G(3d,p) is the method/basis set combination of choice. Initially, all structures have been geometrically optimized to a local minimum of the potential energy surface indicated by the absence of imaginary frequencies. The obtained thermal energies, including zero point energy, E_th_, entropy, S, and electronic energies, E_el_, were implemented in the following equation for calculating the Gibbs energies of the modeled Reactions (1–4) in the gas phase corresponding to conditions such as room temperature, T = 298.15 K, and atmosphere pressure, 1 atm, according to [74]:

∆G^1^ = ∆E_el_ + ∆E_th_ − T∆S

where the ∆ symbol stands for the difference between the products and the reagents. The upper index of 1 corresponds to the dielectric constant of the medium in the gas phase. In order to account for the solvation effect in condensed medium, additional single-point computations at the SMD level [75] were performed and further implemented in the equation:

∆G^ε^ = ∆G^1^ + ∆∆G_solv_ ^ε^ = ∆G^1^ + ∆G_solv_^ε^(products) − ∆G_solv_^ε^(reactants)

where the difference between the condensed-phase and the gas-phase energies of the respective constructs (products–reactants) were used for evaluation of the solvation energies, ∆G_solv_^ε^. Note that it is widely accepted to model protein-rich environments in ε ≈ 4 (emulated by diethyl ether), partially solvent-exposed characterized by ε ≈ 29 (modeled by propanonitrile), and pure aqueous solution with ε = 78. Finally, the ∆G^ε^ of the reaction indicates the susceptibility of the ferric-bound siderophore toward gallium attack depending on the initial Ga^3+^-based complex. The general rule postulates the thermodynamical probability of the reaction with a negative Gibbs energy (successful Ga^3+^→Fe^3+^ substitution), whereas a positive ∆G^ε^ value characterizes a well-protected iron-containing construct. Notably, the present study aims to provide reliable trends and discrepancies affecting the Ga^3+^/Fe^3+^ competition for binding pyochelin and pyoverdine rather than reproducing the absolute numerical values.

The presented figures were prepared through the implementation of the PyMol Molecular Graphics System [76].

### 2.3. Calibration and Validation of the applied DFT Procedure

**Figure 3:**
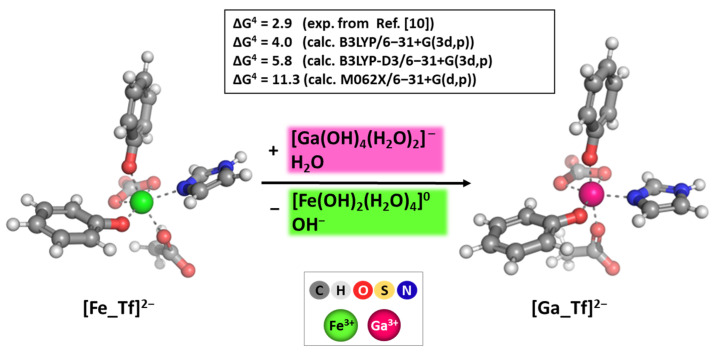
B3LYP optimized structures of Fe^3+^/Ga^3+^-bound ligands from the protein-buried (ε ≈ 4) active center of transferrin and Gibbs energies in kcal mol^−1^ of metal substitution calculated at different theoretical levels in comparison with those experimentally observed and reported in Ref. [45]. The presented color scheme is further applied in Figures 4 to 7.

The applied combination of the method/basis set employed here has been previously implemented and shown to be dependable in delineating the major factors controlling the Ga^3+^/Fe^3+^ competition for binding molecules of biological importance [51,77]. However, its reliability has been further proven for the model reaction of metal substitution enclosed in the protein shell active center in transferrin. The obtained results at three different levels of theory compared to experimentally reported data are presented in the following Figure 3.

Among three different levels of theory, the B3LYP in combination with the double-zeta 6-31+G(3d,p) basis set provided the closest to experiment change in Gibbs energy for metal substitution in the deeply buried surroundings of the active site of transferrin. The obtained result of 4.0 kcal mol^−1^ stays closest to the experimentally observed 2.9 kcal mol^−1^ and within the limitation of the method. The inclusion of empirical dispersion (denoted as B3LYP-D3/6-31+G(3d,p)) and the change of the method to M062X provided ΔG^4^ values that differ substantially from those experimentally observed (5.8 and 11.3 kcal mol^−1^, respectively). Notably, the B3LYP/6-31+G(3d,p) method/basis set, which reliably reproduces the mean bond lengths and, more importantly, the relative difference between the native and abiogenic ions in known X-ray-solved structures of Fe^3+^/Ga^3+^-containing hexaaqua complexes, transferrin, pyochelin, and pyoverdine (in the vicinity of the metal cation), was chosen for executing the present computations (Table 1).

## 3. Results and Discussion

### 3.1. Reactions of Competition with Gallium Nitrate

**Figure 4:**
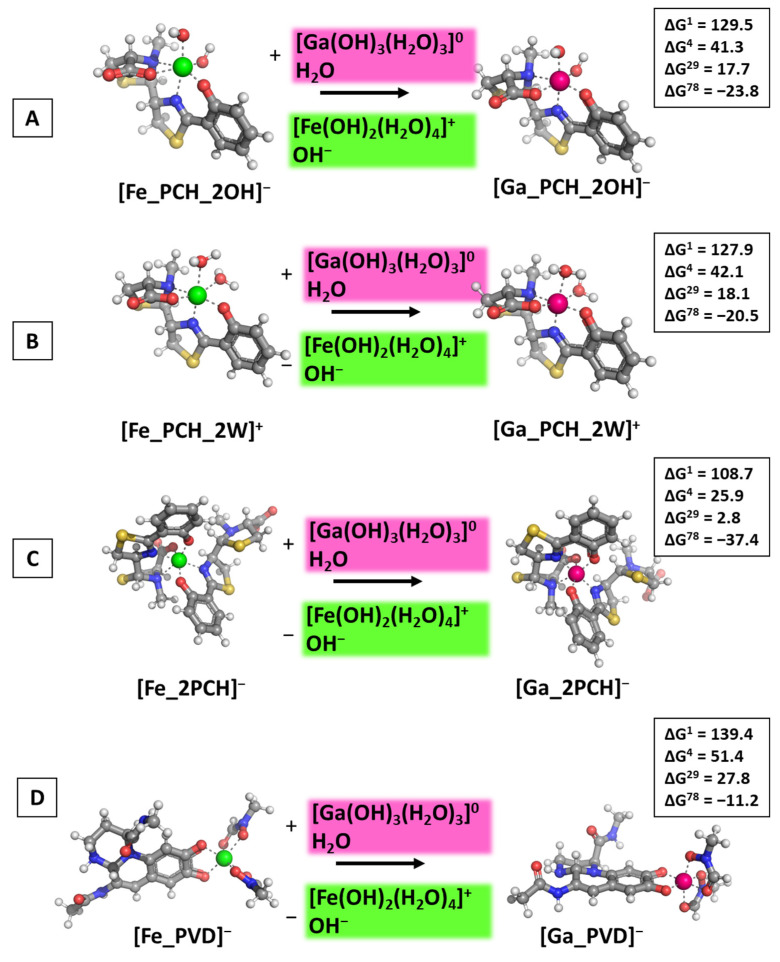
Gibbs energies of metal substitution (in kcal mol^−1^) in four environments of different polarity and acidic pH ≈ 5 in correspondence to the pKa values of the hydrated metal structures optimized at the B3LYP/6-31+G(3d,p) level in [M_PCH_2OH]^−^ (**A**), [M_PCH_2W]^+^ (**B**), [M_2PCH]^−^ (**C**), and [M_PVD]^−^ (**D**). The applied color scheme has been previously presented in Figure 3.

Following reaction R1, the calculations presented in Figure 4 were performed and the obtained results provided.

**Figure 5:**
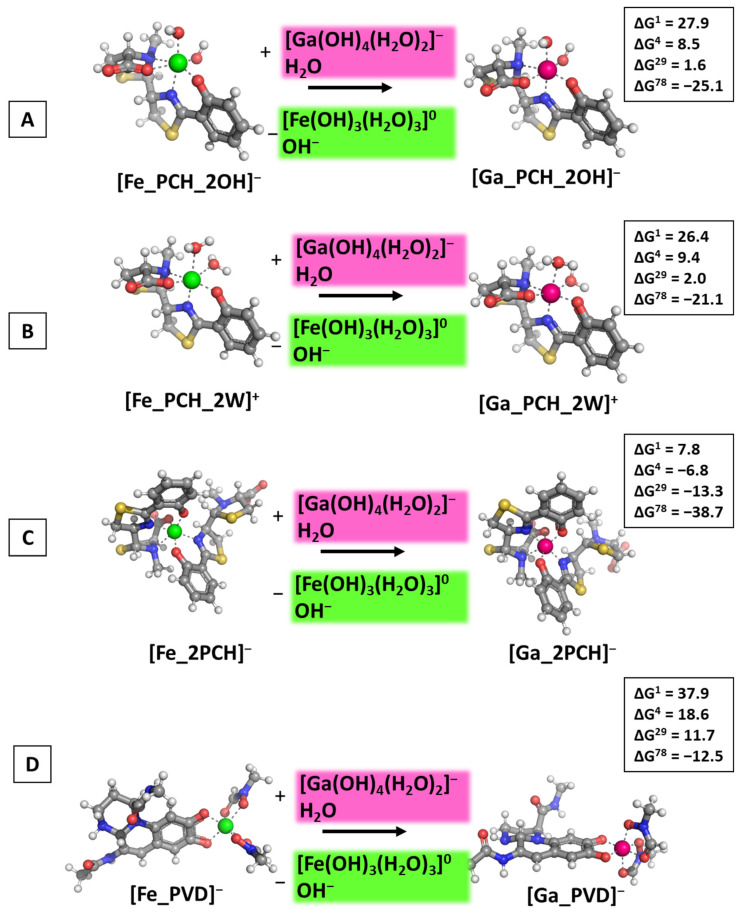
Gibbs energies of metal substitution (in kcal mol^−1^) in four environments of different polarity and physiological pH ≈ 7 in correspondence to the pKa values of the hydrated metal structures optimized at the B3LYP/6-31+G(3d,p) level in [M_PCH_2OH]^−^ (**A**), [M_PCH_2W]^+^ (**B**), [M_2PCH]^−^ (**C**), and [M_PVD]^−^ (**D**). The applied color scheme has been previously presented in Figure 3.

The calculations performed draw a clear picture of the factors controlling the outcome of the metal competition. Firstly, the dielectric constant of the medium, e.g., exposure to solvent plays a crucial role as the reactions become thermodynamically favorable when increasing ε. The abiogenic ion effectively substitutes for the native one in polar aqueous environments, indicated by the negative ΔG^78^ values in all cases ranging from −11.2 to −37.4 kcal mol^−1^ (for the formation of [Ga_PVD]^−^ and [Ga_2PCH]^−^, respectively), while in all other cases (lower dielectric constant) ΔGs stay firmly on positive ground. Interestingly, the type/chemical structure of the siderophore molecule is of greater significance than the additional small ligands—water molecules and hydroxide ions—as the obtained ΔG^ε^ values for the formation of [Ga_PCH_2OH]^−^/[Ga_PCH_2W]^+^ complexes do not differ substantially from each other, whereas the addition of a second pyochelin to the coordination shell of the metal adds approximately 15 kcal mol^−1^ energy gain when the [Ga_2PCH]^−^ construct is formed (Figure 4C). Notably, pyoverdine is the least prone to gallium attack, as evidenced by the highest positive ΔG^ε^ values. Further assessment of the influence of the environment is provided by modeling the attack of Ga^3+^ in neutral (close to physiological) pH in correspondence with the pKa values of the hydrated metal complexes and reaction R2. The obtained results are depicted in Figure 5.

The results obtained indicate that another characteristic of the surrounding medium, pH, also contributes to the outcome in metal exchange. The general following tendencies remain: Gallium outcompetes the native ferric ion, but only when the reactions take place in a polar environment (aquatic environment) as all ΔG^78^ values are all negative; again, in the 1:1 metal:pyochelin constructs the nature of the additional small ligands does not affect gallium’s inability to substitute Fe^3+^ in the non-polar media (positive ΔG^1–29^ values in a close range of each other), although the positive change in the Gibbs energy decreases drastically (by approximately five times) as compared to the same reactions of the formation of the [Ga_PCH_2OH]^−^/[Ga_PCH_2W]^+^ under acidic conditions (examined data presented in Figure 4A,B and Figure 5A,B). Noteworthy, adding a second pyochelin affects the outcome of the metal exchange so strongly that it reverses the previously reported tendency (see above); hence, the alien gallium appears to be able to substitute for the native ferric cation in the [M_2PCH]^−^ construct, even in a protein-rich environment with dielectric constants 4/29 (negative ΔG^4^ and ΔG^29^ values in Figure 5C). It should be emphasized that this result greatly corresponds to the experimentally observed phenomenon that pyochelin does indeed potentiate gallium’s antibacterial effect against *P. aeruginosa* [80]. For the first time (to the best of our knowledge), the calculations presented herewith provide a possible explanation at a molecular level for results reported in literature experiments. Furthermore, the inhibitory effect of pyoverdine could be attributed to gallium’s inability to compete with the native ferric cation, evidenced by the greatest in absolute value positive ΔG^1–29^, especially under acidic conditions, which again falls in line with results reported by Frangipani et.al in Ref. [80]. The comparison between the obtained results at different pH values demonstrates the intriguing revelation that acidic conditions provide additional protection against “alien” attack, giving some explanation as to why *P. aeruginosa* has evolved specific mechanisms to thrive, even at pH ≈ 5 [71].

### 3.2. Reactions of Competition with Gallium Maltolate

**Figure 6:**
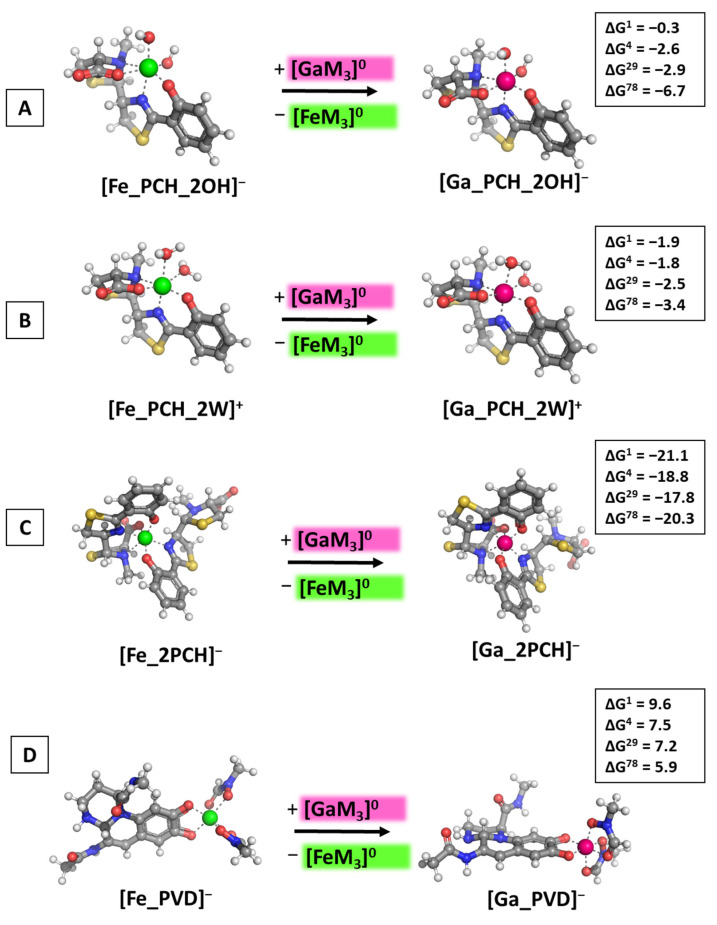
Gibbs energies of metal substitution (in kcal mol^−1^) in four environments of different polarity with an initial gallium-based complex gallium(III) maltolate optimized at the B3LYP/6-31+G(3d,p) level in [M_PCH_2OH]^−^ (**A**), [M_PCH_2W]^+^ (**B**), [M_2PCH]^−^ (**C**), and [M_PVD]^−^ (**D**). The presented color scheme has been previously presented in Figure 3.

The effect of the second-generation gallium-based complex gallium(III) maltolate was assessed by modeling the Ga^3+^-attack in accordance with Reaction 3 (R3). The obtained results are illustrated in Figure 6. 

The computations disclose the intriguing possibility that the maltolate-containing gallium complex displays superior ability to compete with the native ferric cation for binding the pyochelin siderophore in comparison with the hydrated Ga^3+^ complexes as all ΔG^ε^ values remain negative, regardless of the solvent/protein exposure (see Figure 6A–C). The effect of the complementing ligands is observed here as well: the change in Gibbs energies of the formation of the [Ga_PCH_2OH]^−^/[Ga_PCH_2W]^+^ differ slightly by approximately 0.8 kcal mol^−1^, falling within the limits of the error of the method, the only exception being the results in the polar aqueous medium, which can be attributed to the better solvation of the negatively charged hydroxide-containing constructs as compared to their positively charged water-bound equivalents. Furthermore, the presence of a second pyochelin substantially increases gallium’s ability to compete with Fe^3+^, as evidenced by the obtained changes in the Gibbs energies for the formation of [Ga_2PCH]^−^, ranging between −21.1 and −20.3 kcal mol^−1^ (Figure 6C). Note that these are the greatest in absolute value ΔG^ε^, so far indicating that gallium would exert an enhanced therapeutic effect against *P. aeruginosa* when administered in the form of a maltolate as compared to a nitrate (resulting in the formation of a hydrated complex). This observation falls in line with the experimental study by Hijazi et al. [35] reporting that the minimum inhibitory concentration of gallium maltolate is two-times lower than that of the nitrate against three out of four *P. aeruginosa* strains (RPMI-1640/Roswell Park Memorial Institute (RPMI) medium/tissue culture medium supplemented with 10% complement-free human serum). The effect of the dielectric constant of the surrounding medium, however, is much less important in these modeled reactions since the outcome of the metal rivalry does not change with ε: gallium appears able to substitute Fe^3+^ in all pyochelin-containing structures. On the other hand, [Fe_PVD]^−^ is apparently protected against an attack from the abiogenic cation in its maltolate-bound form (ΔG^ε^ values between 9.6 and 5.9 kcal mol^−1^ in Figure 6D). Although the Gibbs energies decrease with enhancing the solvent exposure, they remain strongly positive.

### 3.3. Reactions of Competition with Transferrin

**Figure 7:**
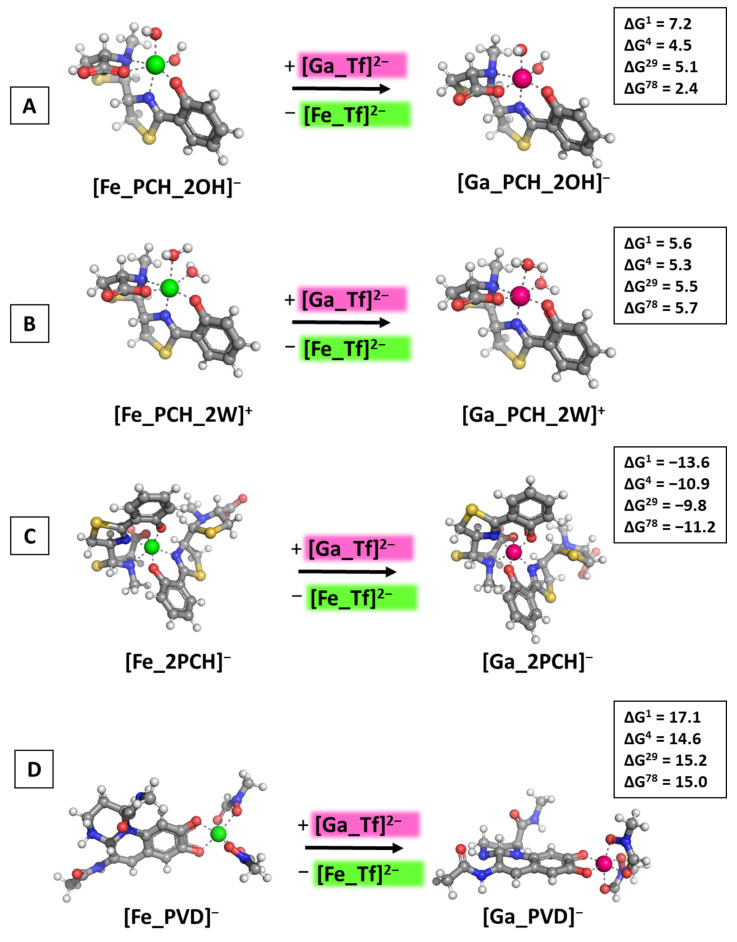
Gibbs energies of metal substitution (in kcal mol^−1^) in four environments of different polarity with an initial gallium-based complex corresponding to the metal binding ligands in the active center of transferrin denoted as [Ga_Tf]^2−^ (explicit structure presented in Figure S1B) optimized at the B3LYP/6-31+G(3d,p) level in [M_PCH_2OH]^−^ (**A**), [M_PCH_2W]^+^ (**B**), [M_2PCH]^−^ (**C**), and [M_PVD]^−^ (**D**). The applied color scheme has been previously presented in Figure 3.

As a final step in the current study, the reactions of Ga^3+^/Fe^3+^ competition for binding the multiple forms of pyochelin and the metal-binding ligands in pyoverdine were modeled with its carrier in the bloodstream transferrin as an initial gallium-based agent. The simplified [Me_Tf]^2−^ constructs were built in the form of metal-ligating groups corresponding to the amino-acid residues in the active center of the protein. The subsequent Figure 7 illustrates the obtained results. 

The presented data once again underline the importance of the structure/properties of the attacking gallium-based complex. Although the previously observed tendencies are also valid for the [Ga_Tf]^2−^ initial construct, there are some thought-provoking insights that should nonetheless be pointed out. Gallium appears able to substitute for the native ferric cation only when two siderophore molecules build the metal coordination shell (negative ΔG^ε^ values for the formation of [Ga_2PCH]^−^ in Figure 7C), while the presence of only one PCH combined with either two hydroxide ions, or two water molecules, leads to a thermodynamically improbable reaction (positive ΔG^ε^ values in Figure 7A,B). This result is unaffected by the change of the dielectric constant, e.g., exposure to the polar aqueous medium unlike the reactions with initial gallium-hydrated complexes [Ga(OH)_3_(H_2_O)_3_]^0^/[Ga(OH)_4_(H_2_O)_2_]^−^, where the computed ΔG^ε^ reverse signs in the water environment. The explanation most probably lies in the necessity of participation of molecules and anions from the surroundings, H_2_O and OH^−^, where the de/solvation energy of the metal constructs strongly depend on the polarity of the medium, especially in the case of the complexes comprising small hydroxide ions. This outcome is further observed for the formation of the pyoverdine-containing structure. The calculated ΔG^ε^ values are all positive, regardless of the dielectric constant of the medium, indicating that [Fe_PVD]^−^ is well protected against gallium outer attack.

## 4. Conclusions

The theoretical study presented herewith delineates physical principles of paramount significance that play a key role in Ga^3+^/Fe^3+^ rivalry for binding the iron-acquiring siderophores pyochelin and pyoverdine secreted by the bacterium *P. aeruginosa*, thus furnishing an (at least partial) explanation of its experimentally observed bacteriostatic effect. Applying the powerful tools of the DFT methodology, we provide proof at a molecular level of gallium’s ability to substitute for the native ferric cation in polar aqueous medium under acidic conditions, and in all simulated dielectric constants at neutral pH when a Fe^3+^ construct in a 1:2 metal:pyochelin ratio is taken into account, thus demonstrating the potentiating effect of PCH, which strongly falls in line with previously reported experimental data. This significant influence of the complementing ligands is further emphasized through comparison with the corresponding water- and hydroxide-containing structures, where gallium appears thermodynamically unable to compete with Fe^3+^ in neither its hydrated form at both pH nor as a transferrin-bound complex if the reaction takes place in a non-polar or partially solvent-exposed medium. The second-generation gallium-based potential drug, gallium maltolate, on the other hand, shows the greatest promise as an iron contender for binding pyochelin in all models in the present study. The obtained ΔG^ε^ values suggest that [GaM_3_]^0^ would exert a better-pronounced therapeutic effect against *P. aeruginosa* in comparison with the other gallium-based complexes, providing a possible mechanism of its action related to its complexation with pyochelin, which has, once again, been observed in the literature. Pyoverdine, on the other hand, exhibits a rather protective function, as it is imune to gallium attack in almost all studied reactions, with the only exception being the reactions in water with hydrated Ga complexes at acidic and neutral pH. Notably, this result is supported by the observation that pyoverdine inhibits Ga^3+^ antibacterial activity. Overall, the performed calculations shed some light on gallium’s ability to compete with the ferric ion for binding the *P. aeruginosa* siderophores pyochelin and pyoverdine and underline the importance of the reaction conditions (acidity and dielectric constant, e.g., exposure to solvent/protein molecules of the surroundings), as well as the nature of the initial Ga^3+^-containing complex and siderophore molecule. Nevertheless, an alternative antibacterial strategy based on metals is still in its infancy, and although the quest from interdisciplinary sciences for forging novel strategies is ongoing, much is yet to be discovered regarding the appealing “Trojan horse” approach.

## Figures and Tables

**Table 1:** Experimental and B3LYP/6-31+G(3d,p) evaluated mean M^3+^–L bond distances (in Å) for Fe^3+^/Ga^3+^ complexes with the ligands under study in the current investigation.

	Fe^3+^		Ga^3+^		Difference R_(Fe^3+^)_ − R_(Ga^3+^)_	
complex	calc	exp	calc	exp	calc	exp
Hexaaqua	2.05	2.00 ^a^	1.96	1.96 ^b^	0.09	0.04
Transferrin	2.07 ^c^	2.03 ^d^	1.98 ^c^	1.98 ^e^	0.09	0.05
Pyochelin	2.18	2.18 ^f^	2.05	1.97 ^g^	0.13	0.21
Pyoverdine	2.07 ^h^	2.11 ^i^	1.98 ^h^	1.95 ^j^	0.09	0.16

^a^ From Ref. [78]. ^b^ From Ref. [79]. ^c^ Modelled as the ligating groups from the protein-buried active center. ^d^ PDB Entry 1D3K. ^e^ PDB Entry 6J2S. ^f^ PDB Entry 1XKW. ^g^ From Ref. [49]. ^h^ Modelled as the metal ligating groups in the vicinity of the M^3+^ cation. ^i^ PDB Entry 2W6T. ^j^ From Ref. [50].

## Data Availability

The data presented in this study are available on request from the corresponding author.

## Supplementary Materials

The following supporting information can be downloaded at: https://www.mdpi.com/article/10.3390/biom14040487/s1, Figure S1: (A) B3LYP/6-31+G(3d,p)-optimized structures of Ga complex with pyochelin (whole molecule) and pyoverdine (a simplified model); (B) B3LYP/6-31+G(3d,p)-optimized structures of gallium(III) nitrate metabolites, gallium(III) maltolate, and a simplified model used for the Ga–transferrin complex.
