# Supplementary material for: In Silico Analysis of the Ga3+/Fe3+ Competition for Binding the Iron-Scavenging Siderophores of P. aeruginosa—Implementation of Three Gallium-Based Complexes in the “Trojan Horse” Antibacterial Strategy
Source: Biomolecules. 2024 Apr 16;14(4):487. doi: 10.3390/biom14040487 (PMC11048449; doi:10.3390/biom14040487)
Supplement: Supplementary file 1 [file biomolecules-14-00487-s001.zip › biomolecules-2950981-supplementary.pdf]

biomolecules

MDPI

Article

In Silico Analysis of the Ga^{3+}/Fe^{3+} Competition for Binding the Iron-Scavenging Siderophores of *P. aeruginosa*—Implementation of Three Gallium-Based Complexes in the "Trojan Horse" Antibacterial Strategy

Nikoleta Kircheva [1], Stefan Dobrev [1], Vladislava Petkova [1], Lyubima Yocheva [2], Silvia Angelova [1,3] and Todor Dudev [2,*]

[1] Institute of Optical Materials and Technologies "Acad. J. Malinowski", Bulgarian Academy of Sciences, 1113 Sofia, Bulgaria; nkircheva@iomt.bas.bg (N.K.); sdobrev@iomt.bas.bg (S.D.); vpetkova@iomt.bas.bg (V.P.); sea@iomt.bas.bg (S.A.)

[2] Faculty of Chemistry and Pharmacy, Sofia University "St. Kliment Ohridski", 1164 Sofia, Bulgaria; lyubima_d_dasheva@abv.bg

[3] University of Chemical Technology and Metallurgy, 1756 Sofia, Bulgaria

* Correspondence: t.dudev@chem.uni-sofia.bg

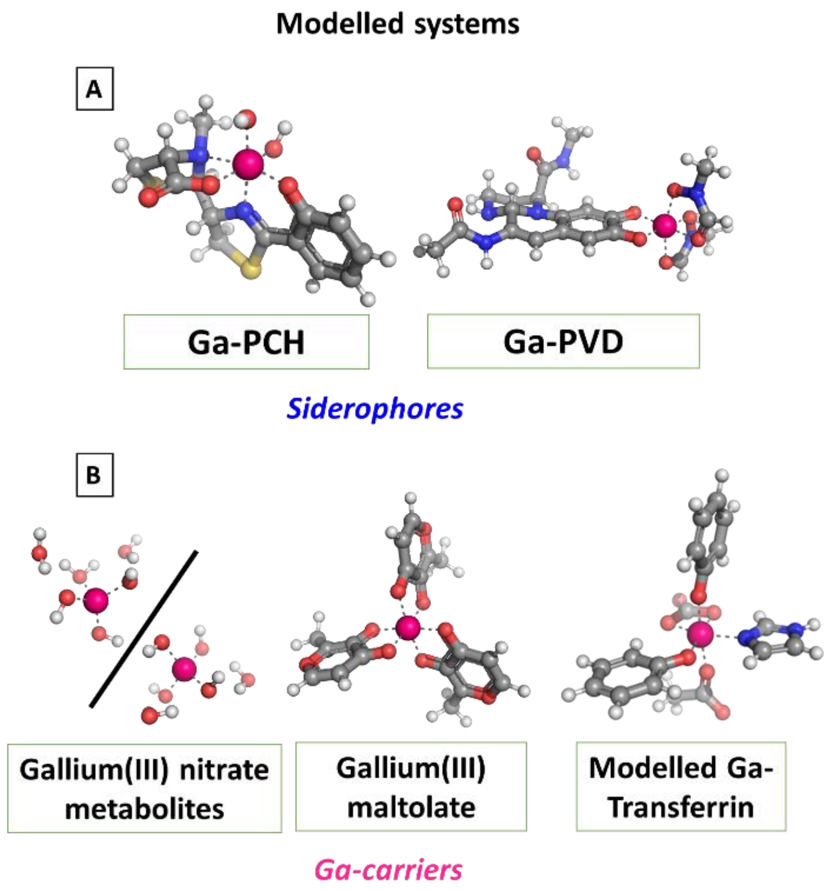

Figure S1. (**A**) B3LYP/6-31+G(3d,p) optimized structures of Ga complex with pyochelin (whole molecule) and pyoverdine (a simplified model); (**B**) B3LYP/6-31+G(3d,p) optimized structures of gallium(III) nitrate metabolites, gallium(III) maltolate, and a simplified model used for the Ga-transferrin complex.
